# Culture-expanded allogenic adipose tissue-derived stem cells attenuate cartilage degeneration in an experimental rat osteoarthritis model

**DOI:** 10.1371/journal.pone.0176107

**Published:** 2017-04-18

**Authors:** Li Mei, Bojiang Shen, Peixue Ling, Shaoying Liu, Jiajun Xue, Fuyan Liu, Huarong Shao, Jianying Chen, Aibin Ma, Xia Liu

**Affiliations:** 1 School of Pharmaceutical Sciences, Shandong University, Jinan, Shandong Province, People’s Republic of China; 2 Post-doctoral Scientific Research Workstation, Shandong Academy of Pharmaceutical Science, Jinan, Shandong Province, People’s Republic of China; 3 Department of Orthopedic Research, Orthopedic Research Institute, St George Hospital University of New South Wales, Sydney, Australia; University of Umeå, SWEDEN

## Abstract

Mesenchymal stem cell (MSC)-based cell therapy is a promising avenue for osteoarthritis (OA) treatment. In the present study, we evaluated the efficacy of intra-articular injections of culture-expanded allogenic adipose tissue-derived stem cells (ADSCs) for the treatment of anterior cruciate ligament transection (ACLT) induced rat OA model. The paracrine effects of major histocompatibility complex (MHC)-unmatched ADSCs on chondrocytes were investigated in vitro. Rats were divided into an OA group that underwent ACLT surgery and a sham-operated group that did not undergo ACLT surgery. Four weeks after surgery mild OA was induced in the OA group. Subsequently, the OA rats were randomly divided into ADSC and control groups. A single dose of 1 × 10^6^ ADSCs suspended in 60 μL phosphate-buffered saline (PBS) was intra-articularly injected into the rats of the ADSC group. The control group received only 60 μL PBS. OA progression was evaluated macroscopically and histologically at 8 and 12 weeks after surgery. ADSC treatment did not cause any adverse local or systemic reactions. The degeneration of articular cartilage was significantly weaker in the ADSC group compared to that in the control group at both 8 and 12 weeks. Chondrocytes were co-cultured with MHC-unmatched ADSCs in trans-wells to assess the paracrine effects of ADSCs on chondrocytes. Co-culture with ADSCs counteracted the IL-1β-induced mRNA upregulation of the extracellular matrix-degrading enzymes MMP-3 and MMP-13 and the pro-inflammatory cytokines TNF-α and IL-6 in chondrocytes. Importantly, ADSCs increased the expression of the anti-inflammatory cytokine IL-10 in chondrocytes. The results of this study indicated that the intra-articular injection of culture-expanded allogenic ADSCs attenuated cartilage degeneration in an experimental rat OA model without inducing any adverse reactions. MHC-unmatched ADSCs protected chondrocytes from inflammatory factor-induced damage. The paracrine effects of ADSCs on OA chondrocytes are at least part of the mechanism by which ADSCs exert their therapeutic activity.

## Introduction

Osteoarthritis (OA) is the most common cause of disability in the elderly and affects approximately 10% of those aged 60 years and older [1]. OA is pathologically characterized by the degeneration of articular cartilage with accompanying inflammatory syndrome and alterations in subchondral bone, and it is clinically characterized by persistent pain, stiffness and disability [2–4]. The epidemiology of OA is multifactorial and complex, with genetic, biological, and biomechanical components [5]. The current therapeutic options for OA include the use of pain medications, nonsteroidal anti-inflammatory drugs (NSAIDs), lubricating supplements, and surgical interventions [6–8]. However, these approaches only focus on temporarily alleviating the symptoms rather than treating the pathogenesis of the disease or reversing the process of OA.

Adipose tissue-derived stem cells (ADSCs) are capable of self-renewing and differentiating into various connective tissue cells, including chondrocytes, osteoblasts, adipocytes and myocytes, under specific induction conditions [9]. ADSCs are abundant and easily acquired by liposuction with minimal donor-site morbidity [10]. It is thought that 1–10% of nucleated cells in adipose tissue are ADSCs, whereas only 0.0001–0.01% of nucleated cells in bone marrow (BM) are stem cells [11]. Moreover, donor age has no influence on the phenotype or function of ADSCs but increased age negatively affects BM mesenchymal stem cells (MSCs) [12]. Recent studies have demonstrated the beneficial effects of ADSCs on OA treatment in animal models [13–15] and clinical trials [16,17]. All clinical trials that have reported the use of ADSCs for the treatment of OA have relied on the use of autologous cells from the stromal vascular fraction (SFV), and most animal studies have also focused on autologous ADSCs. The application of allogenic ADSCs for the treatment of OA is limited. However, the expression of MHC-class-II molecules in ADSCs is low or absent, making it possible to apply allogenic ADSCs for the treatment of degenerative diseases and immune disorders [18]. Additionally, allogenic cells can be a practical ‘off the shelf’ therapeutic agent because they can be isolated from healthy donors and cultured in advance.

In the present study, we investigated the efficacy of the intra-articular injection of culture-expanded allogenic ADSCs in a rat anterior cruciate ligament transection (ACLT)-induced OA model and the paracrine effect of MHC-unmatched ADSCs in chondrocytes in vitro.

## Material and methods

### Animals

Sixty-six male Wistar rats (eight weeks, body weight of 245–255 g, 2 rats for ADSC isolation, 4 rats for chondrocyte isolation and 60 rats for OA treatment) were used for experiments following internationally accredited guidelines with ethics approval from the Institutional Animal Care and Use Committee of the Drug Safety Evaluation Center of Shandong Academy of Pharmaceutical Science (Jinan, China). The rats were housed in groups of 5 per plastic cage on sawdust bedding in a 12:12 light-dark cycle (light-on period, 6:00 AM-6:00 PM) with controlled temperature. They were fed a standard diet and were provided access to filtered water ad libitum. The animals were acclimatized for 1 week before the experiments were started.

### Isolation and characterization of ADSCs

#### Isolation of ADSCs

ADSCs were isolated from adipose tissue from the inguinal regions of the rats according to a previously described method [19]. Primary ADSCs were cultured for 2 weeks according to standard procedures in complete medium [low-glucose Dulbecco’s modified Eagle’s medium (DMEM-LG; Gibco, Gaithersburg, MD, USA) + 10% fetal bovine serum (FBS; Gibco) + 1% penicillin/streptomycin (Gibco)] to 80% confluence. The cells were treated with 0.25% trypsin/0.02 mM EDTA solution for passaging, seeded at a density of 1 × 10^4^ cells/cm^2^, and cultured under the same conditions for expansion (passage 1). Micrographs of the ADSCs were taken with an inverted phase microscope (Nikon TE 2000-U, Tokyo, Japan). Cells at passage 3 were used for the following experiments.

#### Flow cytometry analysis of ADSCs

At passage 3, 1 × 10^5^ ADSCs were suspended in PBS, blocked with rat serum for 30 min and then incubated for 20 min at 4°C in the dark with the following antibodies (Biolegend, San Diego, CA, USA): phycoerythrin (PE)-conjugated anti-CD90, anti-CD45 or anti-CD11b and fluorescein isothiocyanate (FITC)-conjugated anti-CD44. The labeled cells were then analyzed via multiparameter flow cytometry using a FACSCanto cytometer and Diva software (BD-Bioscience, San Jose, CA, USA).

#### Differentiation of ADSCs

To induce adipogenic differentiation, ADSCs at passage 3 were cultured in DMEM-LG supplemented with 10% FBS. After cells reached 70% confluence, the medium was replaced with adipogenic differentiation medium [DMEM-LG supplemented with 10% FBS, 1 μM dexamethasone (Sigma, Bloomington, MN), 10 μM insulin (Sigma), 200 μM indomethacin (Sigma) and 1% penicillin/streptomycin] for an additional 14 days. Adipogenic differentiation was assessed by oil red O staining.

For osteogenic differentiation, the cells were cultured in osteogenic induction medium [DMEM-LG supplemented with 10% FBS, 10 mM β-glycerophosphate (Sigma,), 100 nM dexamethasone, 50 µM ascorbicacid-2-phosphatea (Sigma), and 1% penicillin/streptomycin] for 14 days. Osteogenic differentiation was detected by alizarin red S staining.

To induce chondrogenic differentiation, cells were pellet-cultured in chondrogenic medium [DMEM-LG containing 1% penicillin/streptomycin, 40 μM L-proline (Sigma), 1% ITS solution (Sigma), 5.35 μM linoleic acid (Sigma), 50 μM ascorbate-2-phosphate, 100 nM dexamethasone, 1.25 μM bovine serum albumin (Sigma), and 10 ng/mL recombinant human TGF-ß3 (R&D Systems, Minneapolis, MN, USA)] for 21 days. Chondrogenesis was detected by immunohistochemistry staining of frozen sections of the pellets using a 1:200 dilution of anti-collagen type II antibody (Santa Cruz Biotechnology, Santa Cruz, CA).

### Induction of experimental rat OA

The ACLT OA model was generated as previously described [20]. Rats were anesthetized with 1% pentobarbital sodium, and the right knee joint was exposed through a medial parapatellar approach. For the rats in the OA group, the patella was dislocated laterally, and the knee was placed in full flexion followed by ACL transection with micro-scissors. A positive anterior drawer test was performed to ensure complete ligament transection. In contrast, the rats in the sham group underwent parapatellar incision, lateral dislocation and exposure of the joint without damage to the ligaments. After relocating the patella, both the capsule and the skin were sutured using Vicryl 4–0 absorbable sutures and monofilament 4–0 Nylon threads. After surgery, the rats were allowed free movement within their cages for 4 weeks to develop OA.

### Intra-articular injection of ADSCs

Four weeks after surgery, the OA rats were randomly divided into an ADSC group (n = 20) and a control group (n = 20). Approximately 1 × 10^6^ culture-expanded allogenic ADSCs suspended in 60 μL PBS were intra-articularly injected into the OA joints of the rats in the ADSC group, whereas the control group received 60 μL PBS without cells.

### Macroscopic analysis

Animals were sacrificed at 8 weeks (10 rats in each group) and 12 weeks (10 rats in each group) after ACLT surgery. The femoral condyle and tibial plateau were collected, and the surfaces of the cartilage were examined macroscopically and photographed using a digital camera. Cartilage lesions were evaluated by two examiners who were blinded to treatments according to a previously described method [21]. The scoring system ranged from 0 to 4, with 0 representing normal and 4 representing the most severe degeneration of cartilage.

### Histological analysis

The dissected distal femurs and proximal tibias were fixed with 10% neutral-buffered formalin after gross morphological examination and subsequently decalcified in a decalcifying solution consisting of 5% formic acid, 8.5% hydrochloric acid and 7% (w/v) aluminum chloride. The samples were then dehydrated, embedded in paraffin, and cut into 5-μm-thick sections. The samples were stained with hematoxylin and eosin (H&E) or Safranin-O/fast green via standard procedures. Two examiners blinded to the treatment groups evaluated the severity of cartilage degradation using a modified Mankin scoring system [22]. The evaluation parameters were as follows: 1) cartilage structure (0–6), 2) cartilage cells (0–3), 3) Safranin-O/Fast Green staining (0–4), and 4) tidemark integrity (0–1).

### Isolation and identification of chondrocytes

Chondrocytes were isolated according to a previously described method [23]. Briefly, cartilage slices were harvested from the knee joints of Wistar rats (n = 4) and cut into small pieces. Cartilage pieces were digested with 0.25% trypsin for 30 min at 37°C and then digested with 0.15% type II collagenase for 4 h at 37°C. Chondrocytes were collected by centrifugation (1,200 r/min), suspended in culture medium [DMEM/F12 supplemented with 10% FBS and 1% penicillin/streptomycin], and cultured in 6-well plates in a CO_2_ incubator at 37°C with 5% CO_2_ and maximum humidity. Chondrocytes were passaged upon reaching 80% confluence and used at passage 1 for assays. The morphology of the chondrocytes was observed using an inverted phase microscope, and chondrocytes were identified by the immunocytochemistry staining of type II collagen.

### Co-culture of ADSCs and chondrocytes

Chondrocytes were plated into 6-well plates at a density of 1.5 × 10^4^ /mL and cultured for 24 h. The culture medium was then refreshed with DMEM/F12 containing 20 ng/mL IL-1βto establish OA chondrocytes for a further 24 h in the experimental group. OA chondrocytes were co-cultured with transwell inserts containing 1.5 × 10^4^ ADSCs; for the negative control group, OA chondrocytes were co-cultured with inserts containing 1.5 × 10^4^ chondrocytes. The cells were cultured in 3 mL DMEM/F12 supplemented with 5% FBS, 1% penicillin/streptomycin and 20 ng/mL IL-1β. Mono-cultured chondrocytes not treated with IL-1β served as a positive control. After culturing for 7 days, the mono-cultured and co-cultured chondrocytes were detached using a trypsin solution. Total RNA was extracted from the chondrocytes for quantitative real-time reverse transcription-polymerase chain reaction (real-time PCR) analysis.

### Real-time PCR

Total RNA was extracted using an RNAfast200 RNA extraction kit (Fastagen, Shanghai, China) according to the manufacturer’s instructions. RNA (1 μg) was reverse transcribed into cDNA using a Fast Quant RT Kit (Toyobo Life Science). Primers were designed using Vector NTI (Table 1). Real-time PCR assays were performed using a Roche Light Cycler^TM^ with SYBR Green Real-time PCR Master Mix. The thermal cycling profile of the PCR reaction was as follows: 95°C for 30 s; 40 cycles of 95°C for 5 s, 57°C for 10 s; and 72°C for 15 s. All values were normalized to the housekeeping gene GAPDH, and relative gene expression levels were calculated using the 2^−ΔΔCt^ method, where ΔΔCt = (Ct _target gene_ - Ct_ß-action_)_treated groups_ - (Ct _target gene_ - Ct_ß-action_)_normal_.

**Table 1:** Sequences of primers used in real-time PCR analysis.

Genes	GenBank accession number	Forward primer	Reverse primer
*GADPH*	NM_017008	ACCACAGTCCATGCCATCAC	TCCACCACCCTGTTGCTGTA
*MMP-3*	NM_133523	TCCCTCTATGGACCTCCCAC	TGTTGGATGGAAGAGACGGC
*MMP-13*	NM_133530	AGAAGTGTGACCCAGCCCTA	TCTCGGGATGGATGCTCGTA
*TNF-α*	NM_012675	CACGGAAAGCATGATCCGAG	TCCTCCTTGTTGGGACCGAT
*IL-6*	NM_012509	GCCTTCTTGGGACTGATGTTG	GGAGAGCATTGGAAGTTGGG
*IL-10*	NM_012854	CCTGGTAGAAGTGATGCCCC	TCATGGCCTTGTAGACACCT

### Data analysis

Statistical analysis was performed using the Statistical Package for the Social Sciences (SPSS Inc., Chicago, IL, USA) software version 15.0. All data are presented as the means ± standard deviation (SD). Data for morphological and histological scores were analyzed by Student’s t test. Data from in vitro studies were analyzed by one-way analysis of variance (ANOVA) followed by Bonferroni’s posttest. A value of P < 0.05 was regarded as statistically significant.

## Results

### Phenotype and identification of ADSCs and chondrocytes

**Fig 1:**
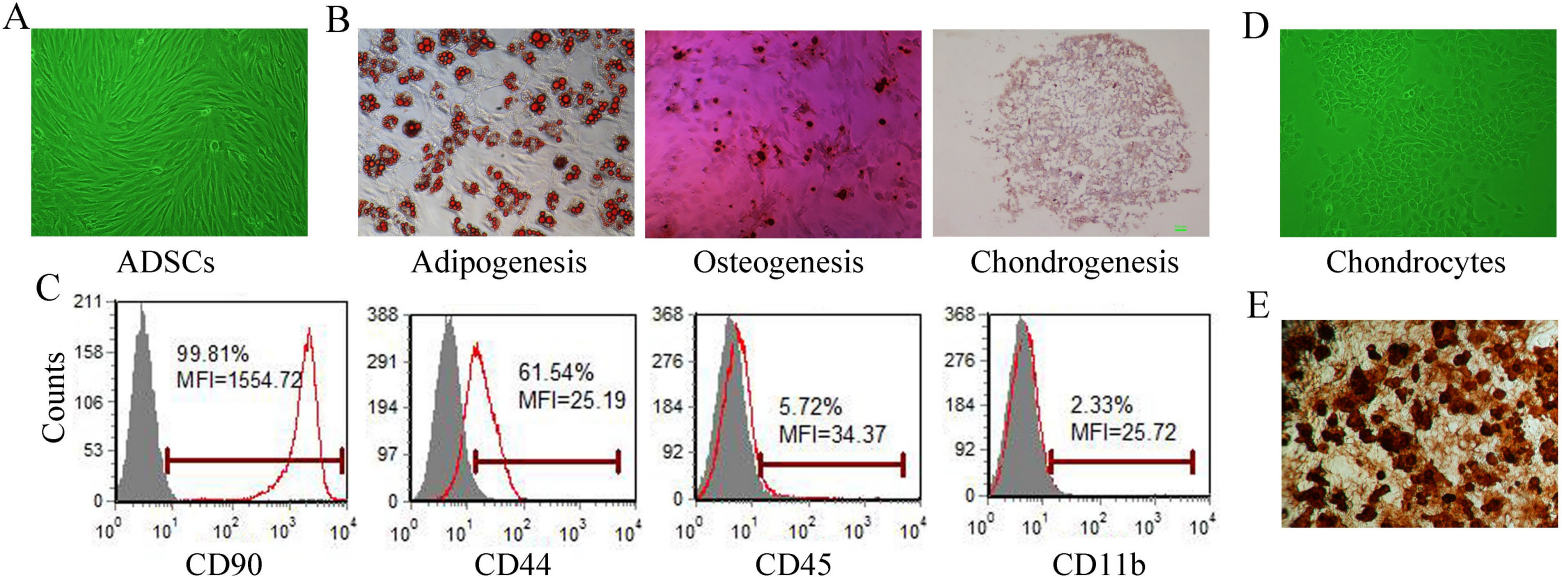
Cell morphology and characterization. Morphology of rat ADSCs at passage 3. (A). Differentiation potential of ADSCs at passage 3. Adipogenic differentiation was detected by positive staining of cytoplasmic lipid droplets with oil red O. Osteogenic differentiation was detected by the appearance of mineralized nodular structures and alizarin red S staining. Chondrogenic differentiation was detected by type II collagen positive staining of the pellet section (B). The immunophenotype of ADSCs at passage 3 was analyzed by flow cytometry for CD45, CD11b, CD90 and CD44 expression (C). Morphology of rat primary chondrocytes (D) and positive staining for type II collagen by immunocytochemistry (E).

The ADSCs exhibited a typical fibroblast-like morphology (Fig 1A). Phenotypic analysis via flow cytometry demonstrated that the ADSCs at passage 3 were positive for the MSC markers CD90 and CD44 and negative for the hematopoietic lineage markers CD45 and CD11b (Fig 1C) and that the ADSCs displayed differentiation potential (Fig 1B). When cultured in adipogenic medium, cytoplasmic lipid droplets of the ADSCs were positively stained with oil red O. The osteogenic induction of ADSCs resulted in positive staining by alizarin red S. Chondrogenic induction resulted in increased expression of type II collagen, as indicated by immunostaining. The chondrocytes exhibited polygon or shuttle shapes, formed a monolayer in a slabstone shape on the plastic surface (Fig 1D) and showed strong expression of type II collagen (Fig 1E). No positive staining was observed in the undifferentiated control cells in any of the differentiation experiments (data not shown).

### Development of mild-grade osteoarthritis

**Fig 2:**
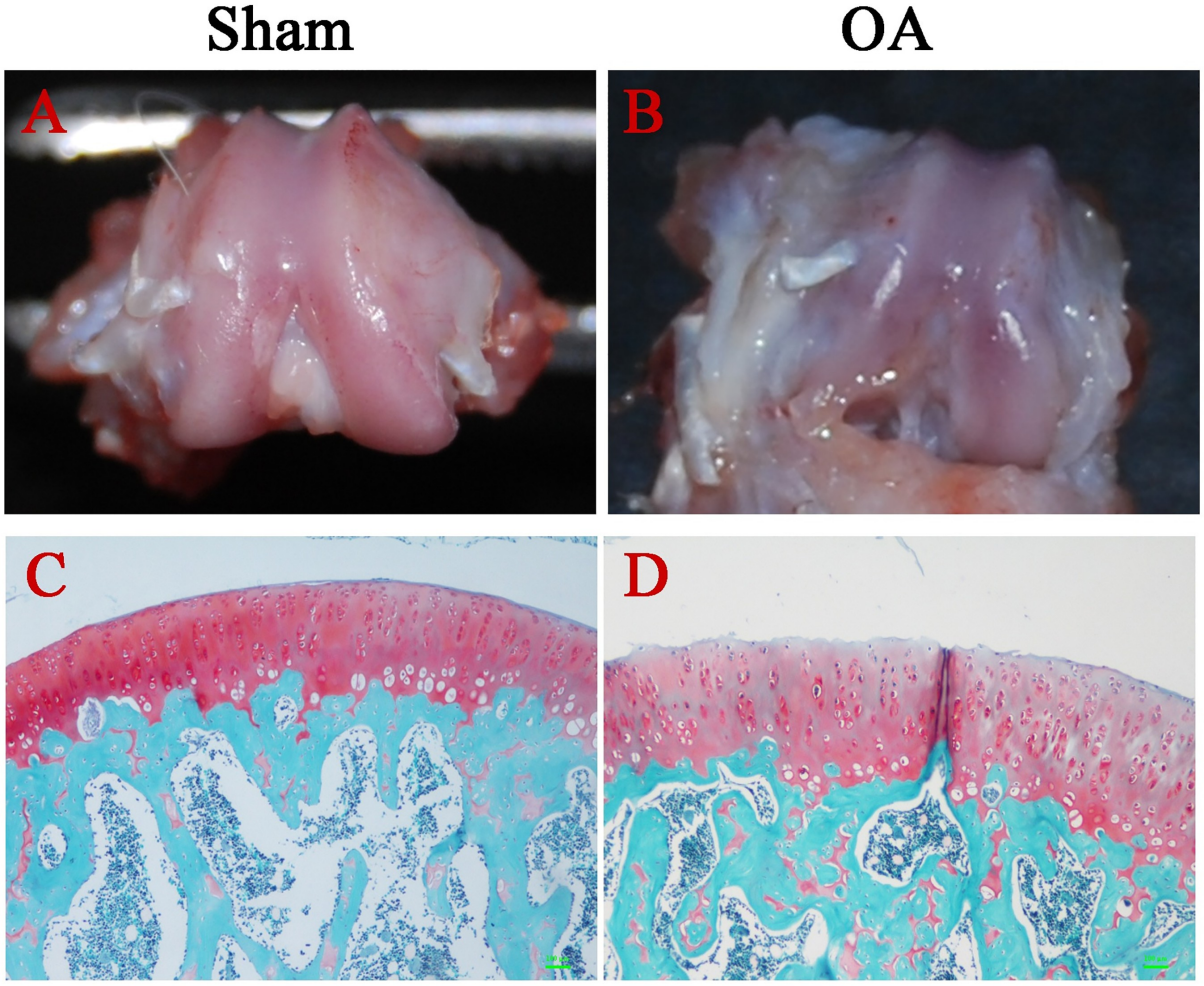
Degenerative changes in articular cartilage 4 weeks after surgery. Gross morphological observation of femoral condyles (A, B) and histological staining (Safranin-O/fast green) of cartilage (C, D).

OA progression was assessed four weeks after surgery. In the OA group, all animals developed mild-grade OA, as evidenced by macroscopic observations and histological staining (Fig 2). Macroscopically, the cartilage surfaces from the animals in the OA group appeared to exhibit moderate erosion and fibrillation. In the sham-operated group, the articular cartilage surfaces were glossy and smooth. The mean macroscopic scores of the OA group were 1.6 ± 0.44 for the femoral condyle and 1.82 ± 0.52 for the tibial plateau, which were significantly higher (P < 0.001) than the values of 0.17 ± 0.1 and 0.2 ± 0.1, respectively, in sham-operated group (Table 2).

**Table 2:** Morphological and histological scores of articular cartilage 4 weeks after surgery.

	Morphological observation		Histological evaluation	
	Femoral condyle	Tibial plateau	Femoral condyle	Tibial plateau
Sham group	0.17 ± 0.10	0.20 ± 0.10	0.30 ± 0.14	0.32 ± 0.12
OA group	1.60 ± 0.44*	1.82 ± 0.50*	5.52 ± 0.10*	6.35 ± 1.27*

Reported as the mean ± SD

* Significantly different between the OA and sham group (P < 0.01).

Histological findings revealed moderate cartilage degeneration with irregular cartilage surfaces and reduced Safranin O staining intensity in the OA group. In the sham-operated group, the superficial area of the cartilage was intact, and the cartilage matrix exhibited intense Safranin O staining. The mean Mankin scores of the OA group (5.52 ± 0.1 for the femoral condyle and 6.35 ± 1.27 for the tibial plateau) were significantly higher (P < 0.001) than the scores of the sham-operated group (0.3 ± 0.14 for the femoral condyle and 0.32 ± 0.12 for the tibial plateau) (Table 2).

### Safety of intra-articular injection of allogenic ADSCs for OA treatment

Behavior, body weight, and food intake were all normal for all experimental rats. ADSC treatment did not cause any local adverse reactions, such as swelling or joint redness. The hematological and hemato-biochemical parameters and histology of the main organs were normal for all experimental rats (data not shown).

### ADSC treatment prevented cartilage degeneration in OA

**Fig 3:**
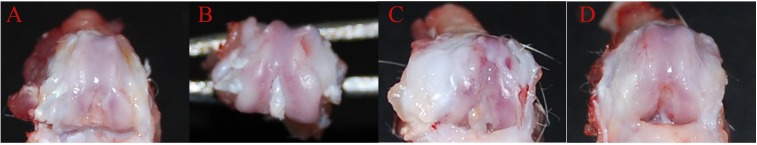
Gross morphological observation of femoral condyles at week 8 and week 12 after surgery. The control group at week 8 (A); the ADSC group at week 8 (B); the control group at week 12 (C); the ADSC group at week 12 (D). Severe erosion (C), moderate erosion (A, D) and mild lesions (B) were observed.

Macroscopic observations of the femoral condyle are presented in Fig 3. Compared to the control group, less erosion and fibrillation of cartilage was observed in the ADSC group at both 8 weeks and 12 weeks after surgery. At week 8, the mean macroscopic scores of the ADSC group were 1.10 ± 0.35 for the femoral condyle and 1.22 ± 0.58 for the tibial plateau, whereas the scores of the control group were 2.30 ± 0.30 for the femoral condyle and 2.37 ± 0.86 for the tibial plateau. At week 12, the scores of the ADSC group were 1.67 ± 0.46 for the femoral condyle and 1.87 ± 0.56 for the tibial plateau, whereas the scores of the control group were 2.98 ± 0.74 for the femoral condyle and 3.20 ± 1.02 for the tibial plateau. Compared with the control group, ADSC treatment significantly decreased the microscopic scores (P<0.05, Table 3).

**Table 3:** Morphological and histological scores of articular cartilage after ADSC treatment.

	Morphological observation		Histological evaluation	
	Femoral condyle	Tibial plateau	Femoral condyle	Tibial plateau
8 weeks				
Control group	2.30 ± 0.30	2.37 ± 0.86	7.10 ± 1.64	8.02 ± 1.28
ADSC group	1.10 ± 0.35 *	1.22 ± 0.58 *	3.85± 1.85 *	4.92 ±1.69 *
12 weeks				
Control group	2.98 ± 0.74	3.20 ± 1.02	9.93 ± 1.81	10.68 ± 2.04
ADSC group	1.67 ± 0.46 *	1.87 ± 0.56 *	6.05 ± 2.20 *	6.18 ± 2.15 *

Reported as the means ± SD

* Significantly different between the ADSC and control group (P < 0.05).

**Fig 4:**
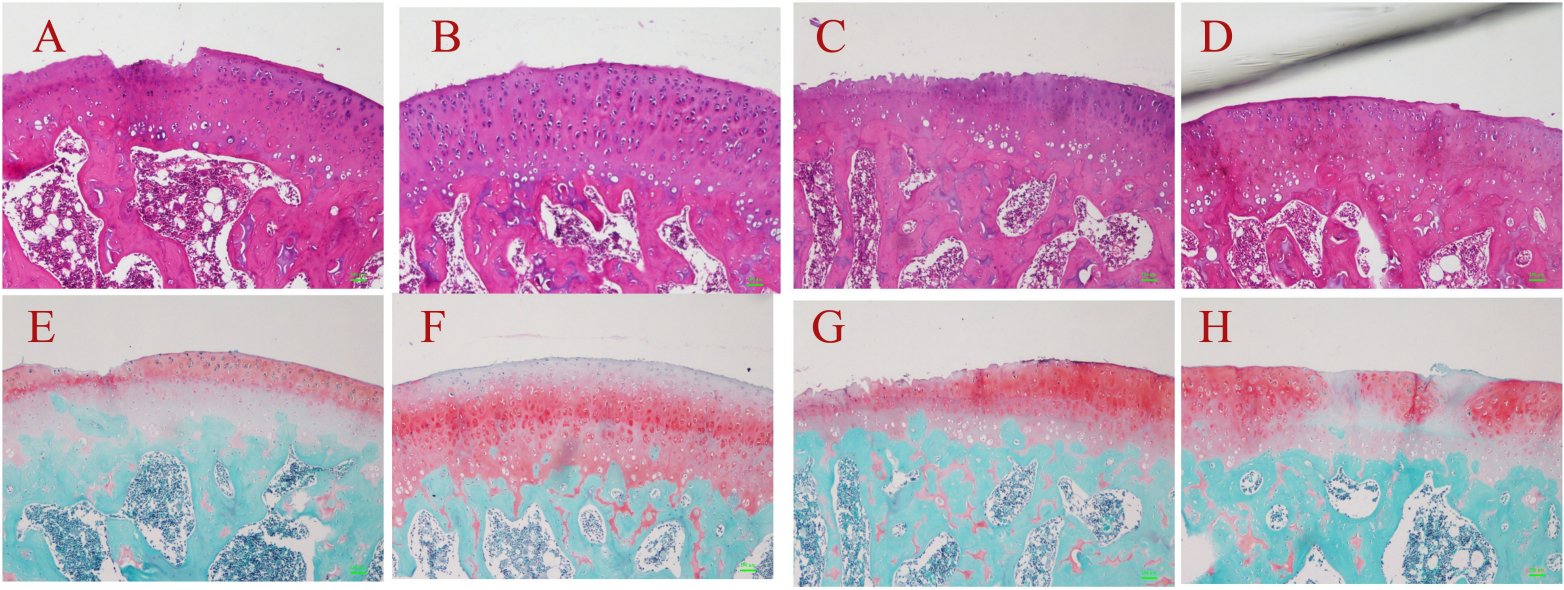
Histological evaluation of femoral condyles at week 8 and week 12 after surgery. H&E staining and Safranin-O/fast green staining of representative specimens. The control group at week 8 (A, E); the ADSC group at week 8 (B, F); the control group at week 12 (C, G); the ADSC group at week 12 (D, H). Severe (C, G), moderate (A, E, D, H) and mild (B, F) cartilage damage were observed.

Histological analyses provided evidence of the protective role of ADSCs on the structure of cartilage tissue in the femoral condyle (Fig 4). Severe cartilage defects and decreased Safranin-O-staining intensity were observed in the control group, whereas fewer cartilage defects and decreased proteoglycan loss were observed in the ADSC group at both week 8 and week 12 after surgery. At week 8, the mean Mankin scores of the ADSC group were 3.85 ± 1.85 for the femoral condyle and 4.92 ± 1.69 for the tibial plateau, and the scores of the control group were 7.10 ± 1.64 for the femoral condyle and 8.02 ± 1.28 for the tibial plateau. At week 12, the ADSC group scores were 6.05 ± 2.20 for the femoral condyle and 6.18 ± 2.15 for the tibial plateau, and the scores of the control group were 9.93 ± 1.81 for the femoral condyle and 10.68 ± 2.04 for the tibial plateau. Compared to the control group, ADSC treatment significantly lowered the Mankin scores (P<0.05, Table 3).

### ADSCs decreased the gene expression levels of matrix-degrading enzymes (MMPs) and inflammatory factors in OA chondrocytes

**Fig 5:**
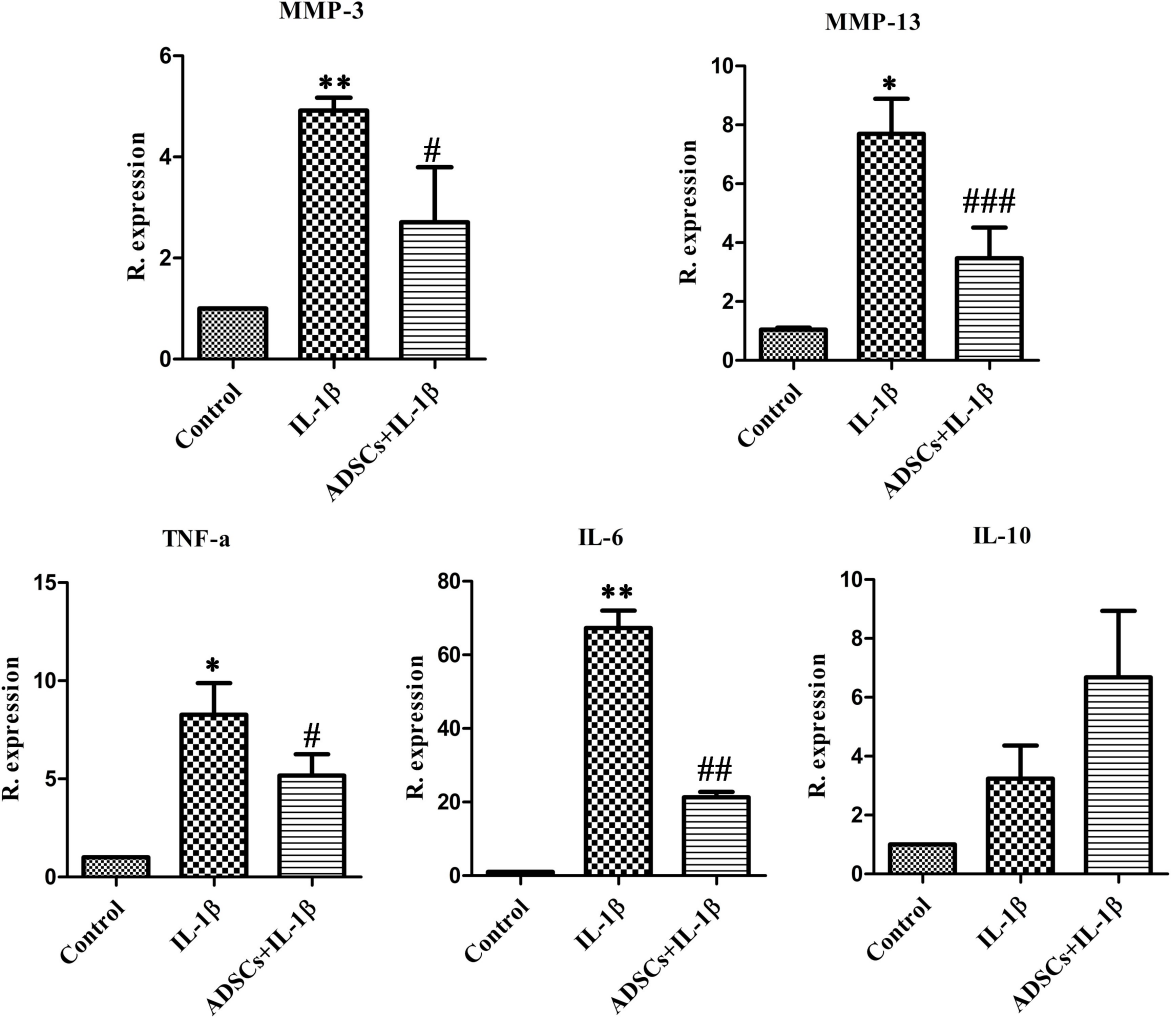
Protective effects of ADSCs on IL-1β-stimulated chondrocytes. The relative mRNA expression levels of MMP-3, MMP-13, TNF-α, IL-6 and IL-10 in chondrocytes were determined by real-time PCR analysis. The data represent the means ± SD. P values < 0.05 for comparisons were considered significant. * P < 0.05 vs the control group, ^#^ P < 0.05 vs the IL-1βgroup.

In the IL-1β-treated group, the mRNA expression levels of the matrix-degrading enzymes MMP-3 and MMP-13 and the pro-inflammatory cytokines TNF-α, IL-6, and IL-10 were significantly increased in chondrocytes compared to those in the untreated control group. Chondrocytes co-cultured with ADSCs showed significantly reduced IL-1β-induced expression of MMP-3, MMP-13, TNF-α, and IL-6, whereas the expression of IL-10 was enhanced, but not significantly (Fig 5).

## Discussion

Stem cell therapy is an emerging option for OA treatment. In this study, we investigated the efficacy of the intra-articular injection of culture-expanded allogenic ADSCs for the treatment of OA in an experimental rat model. We found that allogenic ADSCs did not induce any adverse local or systemic reactions. We generated a rat OA model using ACLT surgery, because the ACLT model mimics various features of human OA and has been widely validated for investigating OA [24]. Four weeks after ACLT surgery, the rats developed a mild degree of OA and were intra-articularly injected with 1 × 10^6^ culture-expanded allogenic ADSCs. Our results showed that, compared with the control group, the ADSC treatment attenuated the ACLT-induced cartilage degeneration based on both macroscopic and histological evaluations performed 8 and 12 weeks after surgery.

Many studies have reported the beneficial effects of autologous ADSCs for the treatment of OA. Toghraie et al. [15] demonstrated that the intra-articular injection of scaffold-free autologous ADSCs improved cartilage quality in an ACLT-induced rabbit OA model. Desando et al. [13] showed that autologous ADSCs prevented damage to cartilage and menisci and attenuated inflammation in synovial membranes in a rabbit OA model. Kuroda et al. [25] found an inhibitory effect of autologous ADSCs on OA progression and showed that the paracrine effects of transplanted ADSCs contribute to this mechanism. Limited studies have evaluated the safety and efficacy of culture-expanded MHC-unmatched allogenic ADSCs for the treatment of OA. Van Pham et al. [26] reported that the intra-articular transplantation of allogenic-unexpanded ADSCs induced the formation of neocartilage in needle disruption-induced cartilage damage in a murine model. In this study, we did not observe the formation of neocartilage in the ADSC group or the control group, but ADSC treatment significantly attenuated cartilage degeneration from OA compared with the control group. The beneficial effect of ADSCs appeared 4 weeks after treatment. After surgery, as time progressed, more serious OA developed in both the ADSC group and the control group. However, the cartilage was thicker in the ADSC group than in the control group. Differences among OA models and treatment times may have led to the different results between our study and previously reported studies.

In this study, the effect of allogenic ADSCs was only evidenced by a reduction in cartilage degeneration, and further studies are needed to improve the efficacy and long-term safety of allogenic ADSCs for the treatment of OA.

There are two possible mechanisms of action underlying the efficacy of stem cells in the treatment of OA. First, transplanted cells differentiate into chondrocytes and fill cartilage lesions. Sato et al. [27] found that direct transplantation of human MSCs into the knee joints of Hartley strain guinea pigs with spontaneous OA could differentiate into chondrocytes and were found to be located in new cartilage. Second, transplanted cells could influence the micro-environment via paracrine actions and by secreting various soluble and insoluble cytokines, chemokines and growth factors to mediate anti-inflammatory, anti-fibrotic, anti-apoptotic and trophic effects in chondrocytes [25,28,29]. Wu et al. [30] reported a beneficial trophic effect of human MSCs on bovine chondrocytes using a pellet co-culture system.

In the present study, we used trans-well co-culture of MHC-unmatched ADSCs and chondrocytes to investigate the paracrine effects of ADSCs on chondrocytes. IL-1β is a major OA inflammatory factor that has been used to induce OA-like changes in vitro [31]. IL-1β stimulation induces chondrocytes to produce catabolic proteases, with apocrine signaling further enhancing the release of MMPs [32]. MMPs make up a large group of extracellular matrix-degrading enzymes that play crucial roles in the initiation and progression of cartilage damage during OA. MMP-3 degrades several macromolecules found in cartilage matrix, including proteoglycans, several collagens and aggrecan link proteins [33]. MMP-13 degrades type II collagen, which is the main component of the cartilage matrix and is responsible for the degradation of native collagen fibers [34]. Our data showed that ADSCs counteracted the upregulation of MMP-3 and MMP-13 in chondrocytes stimulated by IL-1β. Recently, low-grade inflammation has been considered a factor in the OA process. The expression of pro-inflammatory cytokine TNF-α contributes to the subsequent catabolic degenerative processes of OA and stimulates the production of other pro-inflammatory cytokines [35]. Our study revealed that co-culture with ADSCs downregulated the pro-inflammatory cytokines TNF-α and IL-6 but upregulated the anti-inflammatory cytokine IL-10 in IL-1ß-stimulated chondrocytes. These results suggested that MHC-unmatched allogeneic ADSCs may secrete soluble cytokines to protect chondrocytes from inflammatory factor-induced damage.

At the early stage of ADSC treatment of OA, we suggest that the paracrine effect of ADSCs is the main mechanism responsible for their chondro-protective effect. Compared to pre-differentiated ADSCs, direct injection of undifferentiated ADSCs may be advantageous for secreting many factors that mediate diverse functions [36]. As time progresses, the expression levels of chondrogenic genes in ADSCs will increase, and the production of extra-cellular matrix will play a dominating chondro-protective role and induce cartilage reparative effects. Furthermore, the paracrine effect of ADSCs will become weaker. Further studies are needed to validate this hypothesis.

## Conclusion

Intra-articular injection of culture-expanded allogenic ADSCs attenuated cartilage degeneration in an experimental rat OA model without inducing any adverse effects. MHC-unmatched ADSCs protected chondrocytes from inflammatory factor-induced damage. Therefore, the paracrine effect of ADSCs on chondrocytes at least partially contributes to the therapeutic action of ADSCs in OA. However, the action mechanisms of ADSCs in OA need to be further clarified in future studies. Further efforts are also required to determine the potential immune issues associated with allogeneic transplantation of ADSCs and to monitor the fates of transplanted ADSCs.

## Supporting information

S1 File Morphological and histological scores of articular cartilage 4 weeks after surgery. Data for Table 2. Significantly different between the OA and sham group (P < 0.05).
(XLSX)

S2 File Morphological and histological scores of articular cartilage after ADSC treatment. Data for Table 3. Significantly different between the ADSC and control group (P < 0.05).
(XLSX)

S3 File Protective effects of ADSCs on IL-1β-stimulated chondrocytes. Data for Fig 5. The relative mRNA expression levels of MMP-3, MMP-13, TNF-α, IL-6 and IL-10 in chondrocytes were determined by real-time PCR analysis. The data represent the means ± SD. P values < 0.05 for comparisons were considered significant. * P < 0.05 vs the control group, ^#^ P < 0.05 vs the IL-1βgroup.
(XLSX)
